# Clinical application of esophageal manometry: how I do it

**DOI:** 10.1186/s13054-020-03453-w

**Published:** 2021-01-05

**Authors:** Elias Baedorf Kassis, Daniel Talmor

**Affiliations:** 1 Division of Pulmonary and Critical Care and Harvard Medical School, Beth Israel Deaconess Medical Center, Boston, MA 02215 USA; 2 Department of Anesthesia, Critical Care and Pain Medicine and Harvard Medical School, Beth Israel Deaconess Medical Center, Boston, MA 02215 USA

Our group uses esophageal manometry routinely to personalize mechanical ventilation in patients with acute respiratory distress syndrome (ARDS) [1, 2]. Esophageal pressures (Pes) allow for differentiation of chest wall, lung and respiratory system mechanics, and we use this for PEEP titration [1, 2], monitoring of parenchymal lung stress, limiting peak end-inspiratory transpulmonary pressures and monitoring for ventilator synchrony [3, 4].

We find that esophageal manometry is straightforward in the majority of patients although proper training and application are important. The initial step is to assure correct placement with insertion of stand-alone catheters or feeding tubes with integrated esophageal balloons which are similar to routine gastric tubes. Typical depth of insertion ranges from 33 to 40 cm, depending on body size and we assure proper placement through functional bedside assessment. First, we look for the presence of cardiac oscillations to assure correct position posterior to the heart. If absent, this suggests the balloon is too deep or shallow and we incrementally adjust while monitoring for these oscillations. Next we perform expiratory breath holds, with changes in *P*_es_, airway (*P*_ao_) and transpulmonary pressure (*P*_L_ = *P*_ao_ − *P*_es_) monitored during gentle chest pushes. Proper position is confirmed when *P*_es_ and *P*_ao_ increase in equal measure, with no change in the calculated *P*_L_. If *P*_ao_ increases more *P*_es_, this suggests that position is too deep and the balloon is adjusted incrementally with repeat chest pushes. This may be confirmed with gentle abdominal pushes (with *P*_es_ increasing more than *P*_ao_). (Table 1).

**Table 1:** Tricks and troubleshooting

	How we do it	Troubleshooting
Proper placement	(1) Placement depth: Usual depth is 33–40 cm (a good starting point is 37 cm) (2) Balloon inflation: Use a balloon with a consistent working volume. Optimization of volume otherwise will need to be done by measuring the pressure–volume characteristics of the balloon itself which is not always feasible (3) Cardiac oscillations: Cardiac oscillations should be present to confirm placement posterior to the heart above the diaphragm (4) End-Expiratory Hold Chest Pushes: Pes and Pao should increase in equal measures with chest push resulting in no change in *P*_L_	(1) Depth is incrementally adjusted while looking for oscillations and doing chest pushes (2) Depending on body habitus and the unique patient, the amplitude of oscillations may be widely variable (3) If patient is ACTIVELY breathing, expiratory breath holds can still confirm placement. Pao and Pes DECREASES in this case without change in *P*_L_
PEEP titration	(1) Measurement of Pes and PL during expiratory holds (2) Adjust PEEP until PL at end –expiration = zero (3) We use in most patients with moderate-severe ARDS (4) Especially useful with obesity or abdominal hypertension	(1) We no longer use the sliding scale FiO_2_—*P*_L_ used in the EPVent studies, targeting *P*_L_ = zero with acceptable range from -2 to + 2 cmH2O
Monitoring cyclic and total lung stress	(1) Measure end-inspiratory PL: This measurement is obtained when the plateau pressure is measured during an inspiratory breath hold. We keep the end-inspiratory PL < 20cmH2O and ideally aim for < 15cmH2O to provide additional safety (2) Measure ∆*P*_L_: Calculated as the end-inspiratory PL minus the end expiratory PL. This provides a more targeted driving pressure measurement than the respiratory system values, and we aim for less than 10–12cmH_2_O (3) Targeted titration of tidal volume if above target values if allowable with ventilation requirements	(1) With large cardiac oscillations, use the diastole phase for measurements to be consistent (2) We recommend PEEP titration/optimization to maximize compliance prior to targeted tidal volume reduction
Dyssynchrony and neuromuscular blockade	(1) This is easiest with systems that integrate xy plots (2) This is more advanced level application and beyond routine use as above	(1) Not recommended for routine use as requires more specialty equipment and training

Using a balloon with a consistent working range of inflation volume is helpful for obtaining consistent and accurate measurements. While optimal inflation volume can be confirmed based upon the pressure–volume characteristics of the balloon itself [5], this is time-consuming and not required in practice when using a balloon with a known acceptable range. Overinflation results in inaccurately high measured pressures secondary to the compliance of the balloon, while underinflation causes dampening of waveform variation. Visualization and interpretation of data are facilitated by integrated pressure sensors within the ventilator or can be recorded using stand-alone devices as we used in the EPVent and EPVent2 studies [1, 2].

One of our primary applications of esophageal manometry is for titration of positive end-expiratory pressure (PEEP). Critically ill patients frequently exhibit increased chest wall weight and elevated basal end-expiratory pleural pressures secondary to edema, effusions, abdominal hypertension and other causes that may lead to derecruitment, increased lung elastance and hypoxemia. We measure *P*_es_ as a surrogate for pleural pressure [6] and if the pleural pressure is larger than the measured airway/alveolar pressure (PL = Pao − Pes), these collapsing pressures can be countered with the application of PEEP. Our EPVent [2] and EPvent2 [1] studies investigated the use of esophageal manometry to titrate PEEP and while the latter study did not show clear benefit compared with empiric high-PEEP, further analysis suggested a benefit when end-expiratory *P*_L_ were maintained in a tight physiological range of − 2 to + 2cmH_2_O with PEEP adjustment (publication under review) which is how we practice clinically. We aim for an end-expiratory *P*_L_ of zero *regardless of the FiO2* which is distinct from the original sliding-scale protocols [1, 2] and is in part secondary to the slight benefit gained from mediastinal artifact in the “actual” vs. measured *P*_L_ [7].

We find esophageal manometry particularly useful with morbid obesity and ARDS [8, 9], allowing for measurement of elevated pleural pressures and safe application of high PEEP levels (~ 20–30cmH_2_O) to offload the weight of the chest wall. Conversely, esophageal manometry is also useful in determining when applied PEEP is *too high* allowing for targeted titration to lower PEEP which may prevent the harmful effects of overdistension (Fig. 1).

**Figure 1:**
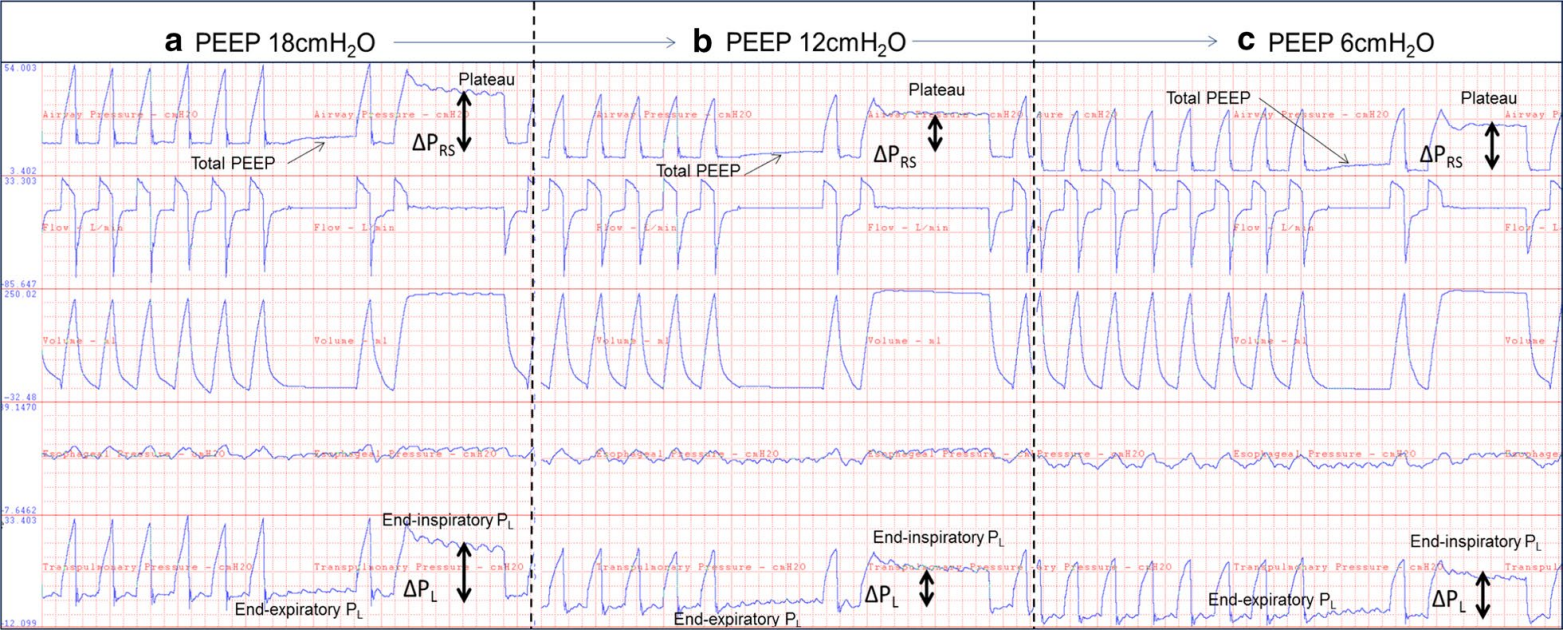
This figure represents positive end-expiratory pressure (PEEP) titration in a mildly obese woman with moderate-severe acute respiratory distress syndrome (ARDS) secondary to ascites and TRALI, with increased chest wall stiffness and mildly elevated basal pleural pressures. Patient was on Vt 250 cc (close to 5 cc/kg IBW), RR 34 and FiO2 0.6. This example illustrates the use in PEEP titration and monitoring of cyclic and total lung stress levels and how esophageal manometry can be used to titrate PEEP not only to HIGH levels, but also be used to titrate downwards to an optimal mid-range. PEEP (airway pressure—*P*_ao_) was adjusted to match the measured esophageal pressure (Pes) to calculate the transpulmonary pressure (*P*_L_ = Pao – Pes) and target a PL equal to zero. **a** Empiric PEEP of 18cmH_2_O (equivalent to using empiric high PEEP ARDSnet tables) was utilized initially on this patient. On these initial settings, the total PEEP was 20cmH_2_O, plateau pressure was 40cmH_2_O, respiratory system driving pressure (∆*P*_RS_) was 20cmH_2_O, and respiratory system compliance was 12.5 ml/cmH_2_O. The end-expiratory transpulmonary pressure (*P*_L_) was + 5cmH_2_O, and end-inspiratory *P*_L_ 20cmH_2_O with a transpulmonary driving pressure (∆*P*_L_) of 15cmH_2_O with a lung compliance of 15 ml/cmH_2_O. These numbers suggested PEEP application was too high and could be resulting in overdistension as measured by the cyclic and total lung stress. **b** Lowering PEEP to 12cmH_2_O resulted in finding optimized mechanics at a PEEP of 12cmH_2_O. This resulted in a total PEEP of 14.5cmH_2_O, plateau pressure of 29.5cmH2O, ∆*P*_RS_ of 15cmH_2_O, respiratory system compliance of 18 ml/cmH2O, end-expiratory PL of + 0.5cmH_2_O, end-inspiratory PL of 11cmH_2_O, ∆*P*_L_ of 10.5 ml/cmH_2_O and lung compliance of 24 ml/cmH_2_O. **c** Dropping PEEP further to 6cmH_2_O resulted in apparent derecruitment with worsened mechanics. Total PEEP was 8.5cmH_2_O, plateau pressure 26.5cmH_2_O, ∆*P*_RS_ 18cmH_2_O, respiratory system compliance 13.9 ml/cmH_2_O, end-expiratory *P*_L_ was − 5.8cmH_2_O, end-inspiratory *P*_L_ was 9cmH_2_O, ∆*P*_L_ 14.8cmH_2_O, lung compliance 16.8 ml/cmH_2_O

As a more specific measure of lung stress, we routinely monitor the cyclic distending pressures across the lungs (transpulmonary driving pressure [∆*P*_L_]) [10]. While the respiratory system driving pressure correlated with mortality in patients with ARDS [11], we believe it is inadequate due to the inherent variability and heterogeneity of the chest wall which we can directly measure using Pes. We target a ∆*P*_L_ of less than 10–12cmH2O due to lung inhomogeneity and local stress raisers [12], which could prevent lung injury [13] and is in agreement with our retrospective mortality data [10]. ∆*P*_L_ is easily measured as the end-inspiratory PL (plateau pressure equivalent) minus the end-expiratory PL (total PEEP equivalent).

In addition to the cyclic lung stress, we use *P*_es_ to measure the total lung stress (end-inspiratory *P*_L_) in addition to plateau pressure. A plateau pressure < 30cmH2O represents a widely varying level of lung stress depending on the chest wall mechanics. While safe levels have not been clearly defined, we have extrapolated thresholds from our understanding of the relationship between stress, strain and specific elastance, and data suggesting high ∆*P*_L_ and end-inspiratory *P*_L_ can bring the lung to total lung capacity and lead to lethal ventilator induced lung injury [14]. As such, our practice is to limit end-inspiratory *P*_L_ to less than 20cmH_2_O (and ideally even lower, to < 15cmH_2_O), to decrease overdistension and improve the margin of safety.

If we identify a patient with elevated ∆*P*_L_ or total end-inspiratory *P*_L_, this data is used to facilitate targeted tidal volume reduction, to bring these values within safer limits. We recognize that further prospective investigation of these limits is warranted to better clarify targets, but we synthesize these data with other clinical data to help inform our bedside care. Importantly, with widely variable chest wall pressures and elastance, *we cannot predict if we are reaching these thresholds* of cyclic and total stress without the use of an esophageal balloon.

We also use esophageal manometry in the bedside assessment of patient–ventilator synchrony using the chest wall pressure volume loops for identification of passive ventilator delivered breaths, spontaneous breaths, dyssynchrony [3, 4], for titration of neuromuscular blockade [6] and for direct measurement of work of breathing, inspiratory muscle efforts and lung-directed mechanical power to assess when levels of effort may be harmful [15]. In conclusion, esophageal balloon catheters are easily placed and interpreted. Measured esophageal pressures and calculation of transpulmonary pressures have broad applications for personalized care of mechanically ventilated patients with PEEP titration, measurement of lung stress and assessment for ventilator synchrony.

## Availability of data and material

Not applicable. If your manuscript does not contain any data, please state not applicable in this section.
